# Metabolic parameters on baseline ^18^F-FDG PET/CT are potential predictive biomarkers for immunotherapy in patients with head and neck squamous cell carcinoma

**DOI:** 10.3389/fmed.2022.896494

**Published:** 2022-09-26

**Authors:** Hye Ryeong Kwon, Junhun Cho, Sehhoon Park, Se-Hoon Lee, Myung-Ju Ahn, Joon Young Choi, Kyung-Han Lee, Hyun Ae Jung, Seung Hwan Moon

**Affiliations:** ^1^Department of Nuclear Medicine, Samsung Medical Center, Sungkyunkwan University School of Medicine, Seoul, South Korea; ^2^Department of Nuclear Medicine, Ilsan Paik Hospital, Inje University College of Medicine, Goyang-si, South Korea; ^3^Department of Pathology, Samsung Medical Center, Sungkyunkwan University School of Medicine, Seoul, South Korea; ^4^Division of Hematology-Oncology, Department of Medicine, Samsung Medical Center, Sungkyunkwan University School of Medicine, Seoul, South Korea

**Keywords:** head and neck squamous cell carcinoma (HNSCC), immune checkpoint inhibitor (ICI), ^18^F-FDG PET/CT, prognosis, PD-L1

## Abstract

**Purpose:**

We evaluated baseline ^18^F-fluorodeoxyglucose (FDG) positron emission tomography/computed tomography (PET/CT) metabolic parameters for predicting prognosis in patients with head and neck squamous cell carcinoma (HNSCC) who were receiving immune checkpoint inhibitors (ICIs). In addition, we also investigated the relationships between immunohistochemical (IHC) biomarkers and metabolic parameters.

**Materials and methods:**

A total of 39 patients with HNSCC who underwent ^18^F-FDG PET/CT prior to ICI therapy between November 2015 and December 2020 were enrolled. PET parameters of tumor lesions included standardized uptake values, metabolic tumor volume (MTV), total lesion glycolysis (TLG), and spleen-to-liver ratio (SLR). Clinical variables, IHC markers, and derived neutrophil-to-lymphocyte ratio (dNLR) were also obtained. Analysis was performed using Cox proportional hazard model, Kaplan-Meier method with log-rank test, and Spearman's correlation.

**Results:**

Total MTV (TMTV), total TLG (TTLG), and a combined parameter consisting of TMTV and dNLR were significant predictors for progression-free survival (PFS) in univariable analysis (TMTV, *p* = 0.018; TTLG, *p* = 0.027; combined parameter, *p* = 0.021). Above all, the combined parameter was an independent prognostic factor for PFS in multivariable analysis. The group with low TMTV and low dNLR had longer PFS than the group with high TMTV and high dNLR (*p* = 0.036). SLR was the only significant predictor for overall survival (*p* = 0.019). Additionally, there was a negative correlation between programmed cell death-ligand 1 expression (one of the IHC markers) and MTV in subgroup analysis.

**Conclusion:**

PET parameters on baseline ^18^F-FDG PET/CT were predictive biomarkers for prognosis in patients with HNSCC undergoing ICI therapy. With dNLR, more accurate prognostic prediction could be possible.

## Introduction

Head and neck cancer accounts for over 9,30,000 new cases and 4,60,000 new deaths annually worldwide (1, 2). The incidence has been on the rise particularly in men, along with the growing importance of this cancer (3). A multimodal approach, consisting of surgery followed by chemoradiotherapy or primary chemoradiotherapy according to the tumor location, is a standard method for treatment. However, treatment for recurrent or metastatic cancer remains a major clinical challenge.

Over the past decade, the introduction of immune checkpoint inhibitors (ICIs) has opened up new opportunities for therapeutic intervention in incurable head and neck cancer (3). Since ipilimumab (a cytotoxic T lymphocyte antigen-4 inhibitor) received Food and Drug Administration (FDA) approval in 2011, the development and application of ICIs have been actively conducted in the field of oncology (4). Pembrolizumab (a programmed cell death-1 inhibitor, a PD-1 inhibitor) alone or in combination therapy improved prognosis compared to standard therapy in programmed cell death-ligand 1 (PD-L1)-positive patients with recurrent or metastatic head and neck squamous cell carcinoma (HNSCC) in a multicenter clinical trial (KEYNOTE-048) (5). ICIs have been recognized as a new and effective treatment option and became a primary treatment for unresectable HNSCC.

The ^18^F-fluorodeoxyglucose (FDG) uptake of HNSCC is generally high and ^18^F-FDG positron emission tomography/computed tomography (PET/CT) has been widely used for staging, response evaluation, and recurrence evaluation (6). There are several studies on the therapy response or prognosis prediction for immunotherapy-treated melanoma and lung cancer using pre-treatment and post-treatment PET/CT (7–10). However, to the best of our knowledge, there were no studies on the usefulness of ^18^F-FDG PET/CT in patients with HNSCC treated with immunotherapy. The baseline metabolic parameters of the primary tumor have a value in predicting prognosis in HNSCC patients who received the standard treatment (11). However, it has not been investigated whether the same conclusion could be reproduced in patients who received immunotherapy. In addition, the correlation between immunohistochemical (IHC) biomarkers related with immunotherapy and PET parameters has not yet been established.

Consequently, we aimed to investigate predictive baseline metabolic parameters for prognosis in patients with HNSCC who were treated with ICIs. We additionally investigated the relationships between IHC markers and PET parameters.

## Materials and methods

### Patient enrollment

We reviewed a total of 89 patients with head and neck cancer who were treated with ICIs between November 2015 and December 2020 in our institution. Among them, 52 patients met the following inclusion criteria: (1) had squamous cell carcinoma; (2) had FDG PET/CT before initiation of immunotherapy; (3) did not undergo surgery or other conventional chemotherapy between the PET/CT and ICI treatment; (4) were without double primary malignancy. The following exclusion criteria were applied to these 52 patients: (1) patients with a time interval of more than 60 days between the date of PET/CT and the date of immunotherapy; (2) those without measurable lesions to be evaluated on PET/CT; (3) those with short-term follow-up loss within 1 month (not due to death). Thirty-nine patients were finally enrolled. This retrospective study was approved by the institutional review board (IRB File No. SMC 2021-02-013) and the need for informed consent was waived.

### Clinical variables

Clinical variables included age, sex, stage, and type of immunotherapy (single regimen or combined regimen). Stage was divided into recurrence group with initial stages 1, 2 or 3, and advanced group with initial stage 4. Furthermore, we obtained serum absolute neutrophil count (ANC) and leukocyte count within 2 weeks from the date of PET/CT. The derived neutrophil-to-lymphocyte ratio (dNLR) was calculated as follows: dNLR = neutrophils/(leukocytes minus neutrophils) (12). The dNLR values were dichotomized by the optimal cut-off obtained from receiver operating characteristic (ROC) curves for progression and death.

Patients underwent neck CT (or MRI) and chest CT scans every 3–6 months to evaluate the therapy response. In some patients, abdominal CT or brain MRI or ^18^F-FDG PET/CT was added depending on the tumor location or new symptoms. Progression was determined by an increment of the lesion size or an appearance of the new lesion, which was confirmed by serial follow-up imaging studies. Progression-free survival (PFS) was defined as the period from the start of immunotherapy to the date of the imaging in which the clue finding was first identified. In the absence of progression, PFS was defined as the period to the date of the last clinical visit. Overall survival (OS) was defined as the period from the start of immunotherapy to the date of death or to the date of the last clinical visit without death. All clinical information was obtained through medical record reviews and imaging reviews.

### ^18^F-FDG PET/CT acquisition

Positron emission tomography/computed tomography images were mostly obtained (27/39 patients, 69%) from a GE STE PET/CT scanner (Milwaukee, WI, USA). Nine cases were obtained from a GE Discovery MIDR PET/CT scanner and three cases from a GE Discovery LS PET/CT scanner. Patients were instructed to fast for at least 6 h and were injected with 5 MBq/kg of ^18^F-FDG. Blood sugar levels of all patients were below 200 mg/dl. After 60 min, a CT scan was performed (STE, 16-slice, 140 keV, 30–170 mA; MIDR, 128-slice, 120 keV, 30–100 mA; LS, 8-slice, 140 keV, 40–120 mA), and a PET scan from the skull base to the thigh was subsequently obtained (STE, 2.5 mm/frame, 3D mode; MIDR, 2 mm/frame, 3D mode; LS, 4 mm/frame, 2D mode). Image reconstruction was performed using an ordered-subsets expectation maximization (OSEM) algorithm for the STE scanner and the LS scanner (2 iterations, 20 subset, matrix size 128 × 128, voxel size 3.9 × 3.9 × 3.3 mm; 2 iterations, 28 subset, matrix size 128 × 128, voxel size 4.3 × 4.3 × 3.9 mm, respectively). OSEM with time-of-flight and point-spread-function was used for reconstruction in the MIDR scanner (4 iterations, 18 subset, matrix size 192 × 192, voxel size 2.6 × 2.6 × 3.3 mm).

### Imaging variables

**Figure 1:**
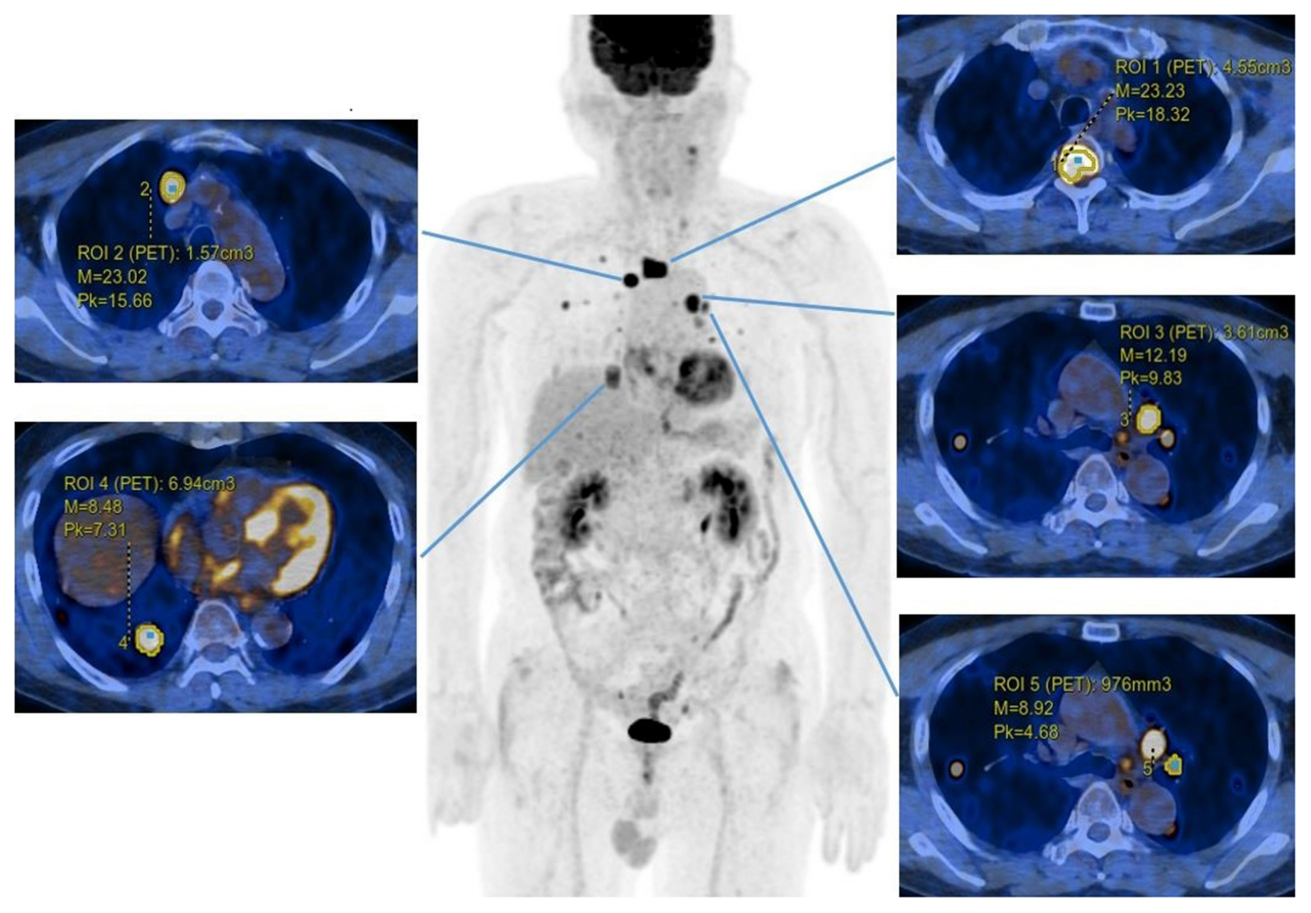
Representative image of lesion measurement. PET parameters were measured in a 71-year-old male patient with multiple metastatic lesions in both lungs, mediastinal lymph nodes, and a bone. The margins within the volume of interests were automatically created according to the 40% threshold of SUV_max_. MTV, SUV_max_, and SUV_peak_ are displayed in the axial fusion images.

One to five tumor lesions were evaluated in each patient according to PERCIST 1.0 criteria (up to two lesions per organ, Figure 1) (13). On a dedicated GE workstation, we set the volume of interest (VOI) of each lesion, which was carefully drawn not to include brain parenchyma. Maximum standardized uptake value (SUV_max_), peak standardized uptake value (SUV_peak_), metabolic tumor volume (MTV), and total lesion glycolysis (TLG) were measured for each lesion. SUV is defined as follows: radioactivity ingested per gram of tissue/radioactivity injected per kilogram of body weight. SUV_max_ represents the highest single-pixel SUV value within the VOI, while SUV_peak_ is an average SUV within a small area containing the hottest uptake and the around. The highest SUV_max_ and SUV_peak_ among all lesions of each patient were selected as representative values for the patient. MTV represents the metabolically active volume by summing the voxels above a threshold. Four thresholds consisting of SUV2.5, 30% of SUV_max_, 40% of SUV_max_, and 50% of SUV_max_ were used for automatically contouring the VOI. TLG is calculated by multiplying MTV and SUV_mean_. Total MTV (TMTV) and total TLG (TTLG) were calculated as the sums of the MTV and TLG of all lesions of each patient. Liver SUV_max_ and spleen SUV_max_ were additionally obtained by setting 3-cm VOIs on the right hepatic lobe and spleen (14). Metastatic lesions were not included within the VOI. Spleen-to-liver ratio (SLR) was calculated by dividing spleen SUV_max_ by liver SUV_max_ (14). The aforementioned PET parameters were dichotomized by optimal cut-off values obtained from ROC curves for progression and death. Combined parameters (MTV + dNLR) were classified into three groups as follows: Group 1, low TMTV and low dNLR; Group 2, low TMTV and high dNLR OR high TMTV and low dNLR; Group 3, high TMTV and high dNLR. Imaging parameters were assessed by two experienced nuclear medicine physicians (SH Moon and HR Kwon).

### IHC biomarkers

Subgroup analysis was performed as a preliminary study on 18 patients with pre-existing PD-L1 (22C3, Dako, Santa Clara, CA, USA) staining results at the primary sites or metastatic lesions (15). Details of the immunohistochemical stains are provided in Supplementary Table 1. The proportion of cells stained with PD-L1 (%) was expressed as a tumor proportion score (TPS) and a combined positive score (CPS). TPS represents the ratio of PD-L1-positive tumor cells among all viable tumor cells, while CPS represents the ratio of PD-L1-positive any cells (including tumor cells, lymphocytes, and macrophages) among all viable tumor cells (15).

Another preliminary analysis was performed on 12 patients with tissues capable of CD8 (SP57, Ventana, Oro Valley, AZ, USA) and granzyme B (11F1, Novocastra, Buffalo Grove, IL, USA) staining. Details of the immunohistochemical stains are provided in Supplementary Table 1. After CD8 and granzyme B staining was performed on formalin-fixed and paraffin-embedded tissue specimens, the ratio (%) of the number of stain-positive cells to the total number of cells was investigated using QuPath software.

The aforementioned procedures were performed by an experienced pathology physician (J Cho). SUV_max_, SUV_peak_, MTV, and TLG of the target lesions were measured to evaluate the association with IHC biomarkers. PET/CT images for this analysis were taken within 1 month from the date of tissue collection.

### Statistical analysis

Survival curves were evaluated for PFS and OS by Kaplan-Meier method and log-rank test. Univariable and multivariable analyses for prognosis were performed using Cox proportional hazard method. Binary PET parameters, combined parameters, and clinical variables were included. Multivariable analysis with enter mode was performed using significant variables of univariable analysis. Multivariable analysis was performed *via* different models for TMTV, TTLG, and combined parameters to avoid multicollinearity. Spearman's correlation was used to evaluate association between immunostaining indicators and PET parameters. IBM SPSS Statistics software (version 27.0) was used, and a *p*-value < 0.05 was considered statistically significant.

## Results

### Clinical characteristics

The characteristics of the subjects are presented in Table 1. Thirty-nine patients with a mean age of 60.7 years and with male predominance (84.6%) were enrolled. Primary tumor sites included nasopharynx (*n* = 6), oropharynx (*n* = 1), hypopharynx (*n* = 11), maxillary sinus (*n* = 2), nasal cavity (*n* = 3), tonsil (*n* = 6), tongue (*n* = 8), and oral mucosa (*n* = 2). Sixteen patients received a single regimen of immunotherapy and 23 patients received a combined regimen. Pembrolizumab (PD-1 inhibitor, 33%, 13/39), nivolumab (PD-1 inhibitor, 36%, 14/39), durvalumab (PD-L1 inhibitor, 28%, 11/39), and avelumab (PD-L1 inhibitor, 3%, 1/39) were used for ICIs. Combined therapies used with ICI included conventional chemotherapy (such as gemcitabine, 5-fluorouracil, and cisplatin), radiotherapy, and proton therapy. Twenty-seven patients (69%) had recurrence and the mean PFS was 238.9 days. These patients were determined to be progressive disease by imaging studies including neck CT (13 cases), neck CT+chest CT (two cases), neck CT+chest CT+abdominal CT (one case), chest CT (six cases), abdominal CT (one case), neck MRI (one case), brain MRI (two cases), and ^18^F-FDG PET/CT (one case). The recurrence sites included neck (13 cases), lung (six cases), neck + lung (two cases), liver (one case), brain (two cases), and bone (three cases). Eleven patients (28%) died and the mean OS was 384.1 days.

**Table 1:** Patient characteristics (*n* = 39).

**Variables**	**No. of patients (%)**	**Mean ± SD**
Age (years)		60.7 ± 14.2 (range, 18–84)
**Sex**		
Male	33 (84.6%)	
Female	6 (15.3%)	
**Stage**		
Recurrence group (Initial stage 1, 2, or 3)	12 (30.8%)	
Advanced group (Initial stage 4)	27 (69.2%)	
**Type of immunotherapy**		
Single regimen	16 (41.0%)	
Combined regimen	23 (58.9%)	
Progression	27 (69.2%)	
Death	11 (28.2%)	
Duration between PET/CT and immunotherapy (days)		20.3 ± 16.1
Progression-free survival (days)		238.9 ± 308.2
Overall survival (days)		384.1 ± 345.7
dNLR		2.8 ± 1.2
SUV_max_		14.3 ± 5.4
SUV_peak_		10.6 ± 4.4
TMTV (cm^3^)		56.1 ± 123.0
TTLG (g)		331.6 ± 564.4
SLR		0.78 ± 0.1

dNLR, derived neutrophil-to-lymphocyte ratio; SUV_max_, maximum standardized uptake value; SUV_peak_, peak standardized uptake value; TMTV, total metabolic tumor volume; TTLG, total total lesion glycolysis; SLR, spleen-to-liver ratio.

### Predictive values of PET and clinical parameters

The cut-off values of PET parameters by each threshold and dNLR were obtained and the results are presented in Supplementary Table 2. Parameters with 40% of SUV_max_ showed the best performance in the analysis, so we decided to describe the findings focusing on this threshold (data with SUV2.5, 30 and 50% thresholds are not shown).

**Figure 2:**
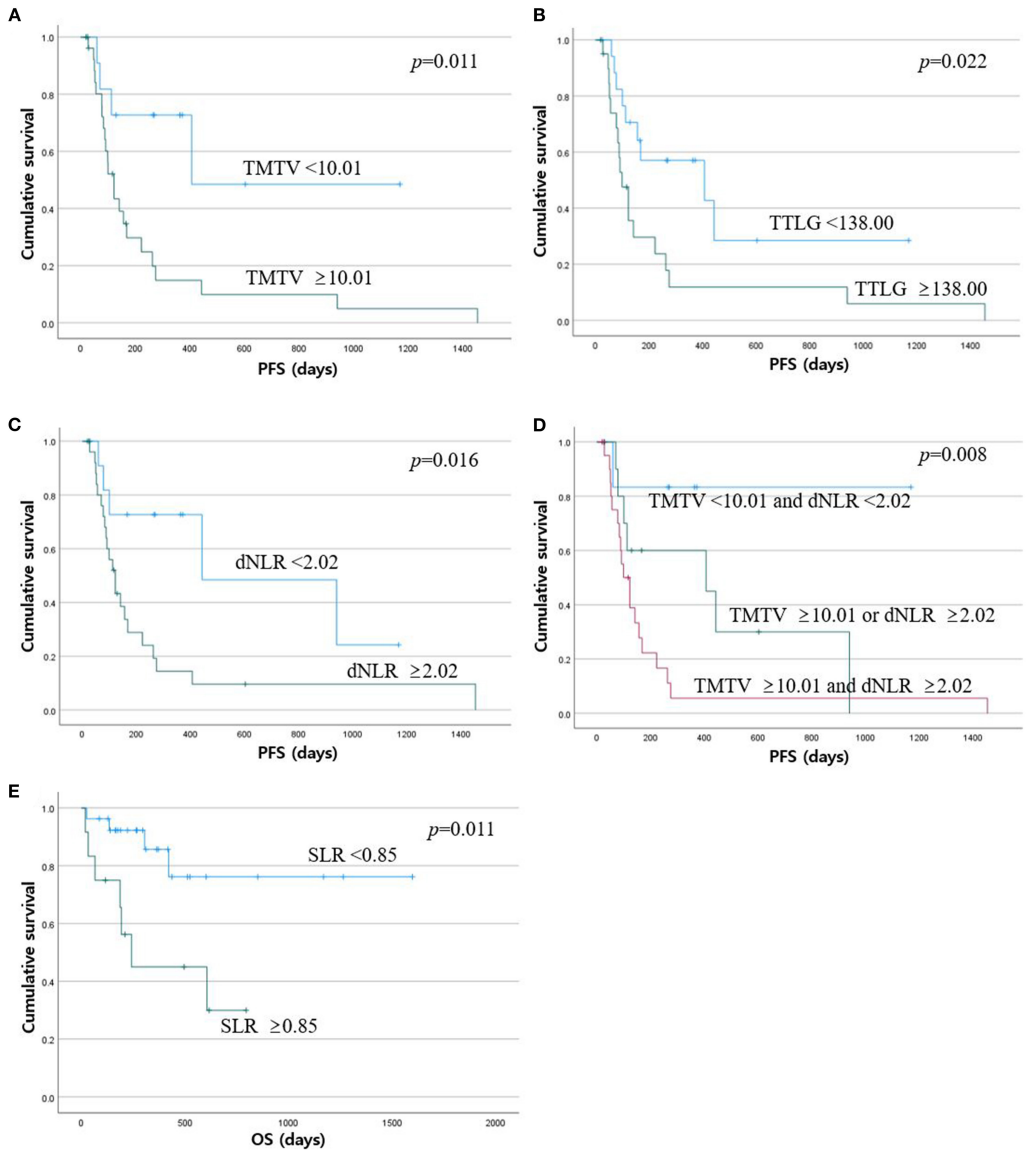
Kaplan-Meier curves with log-rank test for progression-free survival and overall survival. Patients with high TMTV **(A)**, high TTLG **(B)**, and high dNLR **(C)** showed shorter progression-free survival. **(D)** Progression-free survival became shorter from Group 1 to Group 3 for the combined parameter. **(E)** Patients with high SLR had shorter overall survival.

In univariable analysis for PFS using binary PET parameters and clinical variables (Table 2), TMTV (<10.01), TTLG (<138.00), age, dNLR (<2.02), and Group 1 of combined parameters (low TMTV + low dNLR) were significant favorable predictors (*p* = 0.018, *p* = 0.027, *p* = 0.005, *p* = 0.022, and *p* = 0.021, respectively). In multivariable analysis with these variables (Table 3), only the combined parameter and age showed statistical significance. Group 3 had significantly worse PFS than Group 1 [hazard ratio (HR) = 8.91 and *p* = 0.036]. However, Group 1 and Group 2 had no significant difference in progression risk (*p* = 0.116). The older age had a lower risk of progression than the younger age (HR = 0.98 and *p* = 0.048). In Kaplan-Meier curves for PFS (Figure 2), TMTV, TTLG, dNLR, and combined parameters were statistically significant (*p* = 0.011, *p* = 0.022, *p* = 0.016, and *p* = 0.008, respectively).

**Table 2:** Univariable analysis for PFS by Cox proportional hazard model.

**Variables**	**HR (95% CI)**	***p*-Value**
SUV_max_ (<9.74 vs. ≥9.74)	0.62 (0.26–1.51)	0.294
SUV_peak_ (<12.76 vs. ≥12.76)	0.99 (0.45–2.20)	0.982
TMTV (<10.01 vs. ≥10.01)	3.66 (1.25–10.72)	0.018*
TTLG (<138.00 vs. ≥138.00)	2.52 (1.11–5.70)	0.027*
SLR (<0.75 vs. ≥0.75)	1.87 (0.85–4.13)	0.122
Age	0.97 (0.94–0.99)	0.005*
Sex (female vs. male)	0.51 (0.20–1.28)	0.150
Stage (recurrence vs. advanced)	0.89 (0.38–2.08)	0.787
Types of immunotherapy (single vs. combined)	1.13 (0.51–2.51)	0.760
dNLR (<2.02 vs. ≥2.02)	3.17 (1.78–8.55)	0.022*
**Group (TMTV** **+** **dNLR)**		0.026*
Group 1 vs. Group 2	4.90 (0.60–40.15)	0.139
Group 1 vs. Group 3	10.77 (1.42–81.61)	0.021*

PFS, progression-free survival; HR, hazard ratio; CI, confidence interval; SUV_max_, maximum standardized uptake value; SUV_peak_, peak standardized uptake value; TMTV, total metabolic tumor volume; TTLG, total total lesion glycolysis; SLR, spleen-to-liver ratio; dNLR, derived neutrophil-to-lymphocyte ratio; Group 1, low TMTV and low dNLR; Group 2, high TMTV or high dNLR; Group 3, high TMTV and high dNLR;

* , statistically significant.

**Table 3:** Multivariable analysis for PFS by Cox proportional hazard model.

**Variables**	**HR (95% CI)**	***p*-Value**
TMTV (<10.01 vs. ≥10.01)	2.58 (0.84–7.95)	0.100
Age	0.98 (0.95–1.00)	0.064
dNLR (<2.02 vs. ≥2.02)	2.08 (0.72–5.99)	0.176
TTLG (<138.00 vs. ≥138.00)	1.70 (0.69–4.20)	0.251
Age	0.98 (0.95–1.00)	0.037*
dNLR (<2.02 vs. ≥2.02)	2.05 (0.67–6.29)	0.212
**Group (TMTV** **+** **dNLR)**†		0.088
Group 1 vs. Group 2	5.42 (0.66–44.67)	0.116
Group 1 vs. Group 3	8.91 (1.15–68.96)	0.036*
Age	0.98 (0.95–1.00)	0.048*

PFS, progression-free survival; HR, hazard ratio; CI, confidence interval; TMTV, total metabolic tumor volume; dNLR, derived neutrophil-to-lymphocyte ratio; TTLG, total total lesion glycolysis;

* , statistically significant;

† , analysis excluding TMTV, TTLG, and dNLR to avoid multicollinearity.

Spleen-to-liver ratio was the only significant prognostic factor in univariable analysis for OS (Table 4). Patients with high SLRs had a higher risk of death (HR = 4.39 and *p* = 0.019). Similarly, only SLR was statistically significant in survival curve analysis for OS among all PET and clinical parameters (*p* = 0.011, Figure 2).

**Table 4:** Univariable analysis for OS by Cox proportional hazard model.

**Variables**	**HR (95% CI)**	***p*-Value**
SUV_max_ (<16.27 vs. ≥16.27)	0.27 (0.06–1.26)	0.095
SUV_peak_ (<5.35 vs. ≥5.35)	25.88 (0.01–49041.40)	0.398
TMTV (<13.61 vs. ≥13.61)	2.57 (0.55–12.01)	0.230
TTLG (<66.95 vs. ≥66.95)	2.57 (0.55–12.01)	0.230
SLR (<0.85 vs. ≥0.85)	4.39 (1.27–15.10)	0.019*
Age	0.98 (0.94–1.02)	0.402
Sex (female vs. male)	0.50 (0.13–1.90)	0.312
Stage (recurrence vs. advanced)	1.07 (0.28–4.08)	0.922
Type of immunotherapy (single vs. combined)	0.76 (0.23–2.51)	0.655
dNLR (<2.30 vs. ≥2.30)	1.37 (0.36–5.17)	0.645
**Group (TMTV** **+** **dNLR)**		0.583
Group 1 vs. Group 2	1.71 (0.18–16.62)	0.643
Group 1 vs. Group 3	2.68 (0.33–21.90)	0.358

OS, overall survival; HR, hazard ratio; CI, confidence interval; SUV_max_, maximum standardized uptake value; SUV_peak_, peak standardized uptake value; TMTV, total metabolic tumor volume; TTLG, total total lesion glycolysis; SLR, spleen-to-liver ratio; dNLR, derived neutrophil-to-lymphocyte ratio; Group 1, low TMTV and low dNLR; Group 2, high TMTV or high dNLR; Group 3, high TMTV and high dNLR;

* , statistically significant.

### Supplementary analysis using a single PET scanner group and separate immunotherapy regimen groups

To increase the homogeneity of the cohort, the same analysis as above was performed using a group consisting only of the STE PET scanner, which accounts for the majority of study subjects (69.2%, 27/39; Supplementary Table 3). In univariable analysis, TTLG, dNLR, and the combined parameter were significant predictors for PFS (TTLG, HR = 2.80, *p* = 0.037; dNLR, HR = 5.61, *p* = 0.023; TMTV+dNLR, HR = 8.17, *p* = 0.044). Younger age or higher TMTV was more likely to have a progression, but they were not statistically significant (*p* = 0.058 and *p* = 0.062). In multivariable analysis for PFS, Group 3 of the combined parameters was tend to have a higher progression risk than Group 1, but was not statistically significant (*p* = 0.064). There were no statistically significant factors in univariable analysis for OS.

We additionally performed individual analyses for the single regimen group and the combined regimen group with respect to immunotherapy since the treatment type could affect the disease course (Supplementary Tables 4, 5). For single regimen group (*n* = 16), age and the combined parameter were statistically significant in univariable analysis for PFS (HR = 0.94, *p* = 0.026; HR = 10.72, *p* = 0.041, respectively). No significant predictors were found in multivariable analysis for PFS and univariable analysis for OS. For combined regimen group (*n* = 23), TMTV and TTLG were prognostic factors for PFS in univariable analysis (HR = 4.16, *p* = 0.028; HR = 3.24, *p* = 0.044, respectively). There were no independent predictive factors in multivariable analysis for PFS and univariable analysis for OS. SLR showed a marginal significance for OS (*p* = 0.051).

### Association between IHC biomarkers and PET parameters

Spearman's correlation analysis between PD-L1 and PET parameters is presented in Table 5. TPS (%) and MTV showed a moderate negative correlation at all thresholds (MTV2.5, γ = −0.494, *p* = 0.037; MTV30%, γ = −0.619, *p* = 0.006; MTV40%, γ = −0.554, *p* = 0.017; MTV50%, γ = −0.627, *p* = 0.005). CPS (%) and MTV also showed a moderate negative correlation at most of the thresholds (MTV30%, γ = −0.558 *p* = 0.016; MTV40%, γ = −0.487, *p* = 0.040; MTV50%, γ = −0.570, *p* = 0.013). The correlation between TPS and TLG showed a statistical significance only in the threshold of 50% (*p* = 0.045). There was no significant correlation between CPS and TLG. SUVs showed no significant relationships with TPS or CPS.

**Table 5:** Correlation between PD-L1 expression and PET parameters.

**PET parameters with threshold**	**PD-L1 TPS (%)**		**PD-L1 CPS (%)**	
	**Spearman's rho**	***p*-Value**	**Spearman's rho**	***p*-Value**
SUV_max_	−0.126	0.619	−0.015	0.954
SUV_peak_	−0.072	0.775	0.091	0.721
MTV2.5	−0.494	0.037*	−0.420	0.083
MTV30%	−0.619	0.006*	−0.558	0.016*
MTV40%	−0.554	0.017*	−0.487	0.040*
MTV50%	−0.627	0.005*	−0.570	0.013*
TLG2.5	−0.414	0.087	−0.351	0.154
TLG30%	−0.448	0.062	−0.372	0.129
TLG40%	−0.404	0.097	−0.321	0.194
TLG50%	−0.478	0.045*	−0.398	0.102

PD-L1, programmed cell death-ligand 1; TPS, tumor proportion score; CPS, combined positive score; SUV_max_, maximum standardized uptake value; SUV_peak_, peak standardized uptake value; MTV, metabolic tumor volume; TLG, total lesion glycolysis;

* statistically significant.

Regarding the number and activity of cytotoxic T cells, both CD8 and granzyme B biomarkers were not related to metabolic parameters. The *p*-values of SUV_max_, SUV_peak_, MTV40%, and TLG40% for association with CD8 positivity (%) were *p* = 0.557, *p* = 0.681, *p* = 0.542, and *p* = 0.471, respectively. For association with granzyme B positivity (%), the *p*-values were *p* = 0.395, *p* = 0.415, *p* = 0.913, and *p* = 0.595, respectively.

## Discussion

We investigated the prognostic potential of baseline ^18^F-FDG PET/CT in HNSCC treated with ICIs and found that high volumetric metabolic PET parameters were predictive indicators for poor clinical outcome. The higher the TMTV and TTLG, the shorter the PFS. In the conventional cancer treatment, it is already known that high metabolic parameters are unfavorable prognostic indicators (16, 17). Our results support that the same interpretation could be applied to the immunotherapy field. Metabolic tumor burden is still a strong predictive factor regardless of the treatment type. Additionally, this study is the first to evaluate the prognostic value of ^18^F-FDG PET/CT in immunotherapy-treated HNSCC. Some other carcinomas were already evaluated for the usefulness of PET/CT in this field. Castello et al. (18) reported that non-small cell lung cancer (NSCLC) patients receiving ICIs with high MTV/high TLG/high inflammatory index (dNLR and platelet count) had an increased risk of hyperprogressive disease. Ito et al. (8) reported that melanoma patients receiving ipilimumab with high TMTV showed significantly lower median OS and that TMTV was an independent prognostic factor in multivariable analysis. Although lung cancer and melanoma are currently the mainstream in immunotherapy research, the broadened basis is expected to other cancers.

Semiquantitative parameters such as SUV_max_ and SUV_peak_ are generally known to suggest a worse prognosis as their values increase in the conventional therapy. However, there are conflicting opinions on the predictive values of those SUV parameters in the immunotherapy. Some studies suggested that high baseline SUV_max_ in NSCLC is paradoxically related to good ICI response, since immune infiltration promotes glycolytic activity (9, 10, 19). Meanwhile, there was a study reporting that tumor SUV_max_ was not a significant factor for survival in patients with advanced lung cancer receiving ICI, which is similar to ours (12). It is a weak point for SUV in that only the highest single value among several lesions was used as the representative. It is not sufficient to reflect the entire tumor burden. Another disadvantage of SUV is that it is affected not only by malignant tissue but also by inflammatory activity (20). An intense hypermetabolic lesion due to inflammatory cells is unlikely to be associated with a poor outcome, especially in immunotherapy. This study suggested a patient with multiple large lesions of moderate FDG uptake might be more likely to have progression than a patient with one small lesion of high FDG uptake.

We expected a significant relationship between inflammatory markers and immunotherapy response. Absolute neutrophil count, absolute leukocyte count, and dNLR were related to the therapeutic response and prognosis for immunotherapy (12, 21). One paper reported that lower NLR might correlate with disease control and treatment response in patients with advanced lung cancer who received PD-1 inhibitors (22). Another paper reported that high dNLR was associated with no response to nivolumab in patients with NSCLC (23). Castello et al. (18) suggested MTV and dNLR were independent prognostic factors for OS in multivariable analysis in NSCLC patients treated with ICI. High dNLR as a poor prognostic factor was also demonstrated in our study. This appears to be related to tumor T-cell infiltration (24). The dNLR as a serum inflammatory biomarker is likely to secure the predictive value in immunotherapy-treated HNSCC. SLR is an imaging inflammatory marker because of its correlation with serum C-reactive protein level, white blood cell count, and neutrophil count (25). Seban et al. (14) reported that high TMTV, SLR, and bone marrow-to-liver SUV_max_ ratio were associated with lower survival in patients with melanoma receiving ICI. They suggested the potential of hematopoietic tissue metabolism in predicting clinical outcomes for immunotherapy. Similarly, SLR was observed to be statistically significant for OS in this study. The metabolic reversal of spleen compared to liver is usually associated with infectious or inflammatory general conditions, which may affect therapeutic response (26).

Regarding the combined parameters consisting of two predictors, TMTV and dNLR, patients with Group 3 (high in both TMTV and dNLR) had a significantly shorter PFS and higher risk of progression than patients with Group 1 (low in both TMTV and dNLR). This is noteworthy because the respective TMTV and dNLR were not significant in multivariable analysis. Using the PET parameter and dNLR together might result in a more robust prediction. This is consistent with the result from a former study combining MTV and dNLR in lung cancer. Seban et al. (12) reported that TMTV >75 and dNLR >3 were correlated with lower OS and no clinical benefits in advanced NSCLC patients treated with ICI. Interestingly, there was no significant difference between Group 2 (high in either TMTV or dNLR) and Group 1. Group 3 therefore needs more attention for poor prognosis prior to immunotherapy than other groups.

Age was an independent predictive indicator. The prognostic impact of age in head and neck cancer is controversial. A previous study suggested that increasing age was an unfavorable predictor for OS (27). Another study suggested that younger age (<30 years) and older age (>50 years) groups showed higher risks of recurrence than the middle age group (30–50 years) (28). Meanwhile, Gilroy et al. (29) reported that age was not associated with prognosis. We found the progression risk increased at a younger age and it can be assumed to be associated with rapid tumor differentiation and growth at young age.

As a preliminary study, we tried to determine whether PD-L1 expression was predictable by PET parameters. PD-L1 expression of tumors is a key point in the use of ICIs such as PD-L1 inhibitors and PD-1 inhibitors. Several studies have shown that PD-L1 expression is associated with a good prognosis (30, 31). For PET parameters, previous studies mainly investigated the positive correlation between SUV_max_ and PD-L1 in other cancers (32–34). We found PD-L1 was negatively correlated with MTV and not correlated with SUVs. Although we could not find a similar research to ours, it could be hypothesized that the higher the PD-L1 expression, the less tumor growth, leading to a better prognosis. Since this was from a subgroup analysis performed with a much smaller number of patients, further studies are needed.

We also tried to evaluate the predictive PET findings for CD8 and granzyme B. CD8 is a glycoprotein located on the surface of cytotoxic T cells and refers to the number of cytotoxic T cells. These cytotoxic T cells play a key role in cancer immunotherapy (35). Granzyme B is a serine protease found in the granules of cytotoxic T cells. Since CD8 T cells express granzyme B only when they are stimulated and differentiated into cytotoxic lymphocytes, granzyme B reflects the activity of cytotoxic T cells (36). Considering the mechanism of immunotherapy related with cytotoxic T cells, and as there was a correlation between PET parameters and immunotherapy response, we inferred a significant relationship between PET parameters and cytotoxic T cells. However, no significant correlation was found between the variables in this study.

This study has some limitations. First, this was a retrospective study with a small number of patients. Second, PET/CT images of all patients were not obtained with the same scanner, although most of the images were taken with a GE STE scanner. Third, the immunotherapy regimens of patients were not the same. Regarding the limitation of data heterogeneity, we performed supplementary analyses with a single scanner group, a single regimen group, and a combined regimen group. Only some, but not all, parameters which showed significances in the original cohort were statistically significant in each analysis. Although partially matched outcomes were obtained, the results were incomplete. The number of cohorts for each subgroup might be too small to make a conclusion. Therefore, future studies with sufficient number of patients using the same ICI regimen and the same imaging scanner are warranted for more accurate evaluation.

## Conclusion

High total volumetric parameters on baseline ^18^F-FDG PET/CT suggests a high risk of progression in HNSCC patients receiving ICIs. High dNLR also indicates a poor prognosis. Combining these two parameters enables the stratification of progression risk. Patients with high TMTV and high dNLR are more likely to have a poor prognosis, and consequently require more caution in the clinical practice. For IHC markers, there was a negative correlation between PD-L1 expression and MTV. Despite several limitations, this study suggests the prognostic potential of ^18^F-FDG PET/CT in immunotherapy-treated HNSCC.

## Data availability statement

The original contributions presented in the study are included in the article/Supplementary material, further inquiries can be directed to the corresponding authors.

## Ethics statement

The studies involving human participants were reviewed and approved by Institutional Review Board of Samsung Medical Center. Written informed consent for participation was not required for this study in accordance with the national legislation and the institutional requirements.

## Author contributions

All authors listed have made a substantial, direct, and intellectual contribution to the work and approved it for publication.

## Funding

This work was supported by a National Research Foundation of Korea grant funded by the Korean government (No. NRF-2019R1F1A1060353).

## Conflict of interest

The authors declare that the research was conducted in the absence of any commercial or financial relationships that could be construed as a potential conflict of interest.

## Publisher's note

All claims expressed in this article are solely those of the authors and do not necessarily represent those of their affiliated organizations, or those of the publisher, the editors and the reviewers. Any product that may be evaluated in this article, or claim that may be made by its manufacturer, is not guaranteed or endorsed by the publisher.
