# Supplementary material for: Metabolic parameters on baseline 18F-FDG PET/CT are potential predictive biomarkers for immunotherapy in patients with head and neck squamous cell carcinoma
Source: Front Med (Lausanne). 2022 Sep 26;9:896494. doi: 10.3389/fmed.2022.896494 (PMC9548588; doi:10.3389/fmed.2022.896494)
Supplement: Supplementary file 1 [file Data_Sheet_1.docx]

**Supplementary Table 1.** Antibodies used for immunohistochemistry

| **Antibodies (clone)** | | **Cat. No.** | **Clone** | **Company** | **Dilution** | **Antigen retrieval** | **Endogenous peroxidase blocking** | **Incubation time with primary antibody** | **Secondary and chromogen** | **Machine** |
| --- | --- | --- | --- | --- | --- | --- | --- | --- | --- | --- |
| PD-L1 | 22C3 | M3653 | 22C3 | Dako | 1:50 | 64 min with CC1 in Ventana BenchMark Ultra (Standard-CC1) | 4 min | 60 min with Ventana BenchMark Ultra at 37 ℃ | OptiView DAB IHC Detection Kit  (760-700) | BenchMark Ultra |
| CD8 | CONFIRM anti-CD8 (SP57) Rabbit Monoclonal Primary Antibody | 790-4460 | SP57 | Ventana | RTU | 60 min with CC1 in Ventana BenchMark XT (Standard-CC1) | 4 min | 24 min with Ventana BenchMark XT at 37 ℃ | ultraView Universal DAB Detection Kit (760-500) | BenchMark XT |
| Granzyme B | 11F1 | NCL-L-GAN-B | 11F1 | Novocastra | 1:50 | 36 min with CC1 in Ventana BenchMark Ultra (Standard-CC1) | 4 min | 32 min with Ventana BenchMark Ultra at 37 ℃ | ultraView Universal DAB Detection Kit (760-500) | BenchMark Ultra |

**Supplementary Table 2.** Cut-off values of parameters by ROC curve analysis

| Variables | PFS | | | |  | OS | | | |
| --- | --- | --- | --- | --- | --- | --- | --- | --- | --- |
|  | Cut-off | Sen | Spe | AUC |  | Cut-off | Sen | Spe | AUC |
| SUV_max_ | 9.74 | 0.26 | 0.92 | 0.48 |  | 16.27 | 0.82 | 0.46 | 0.43 |
| SUV_peak_ | 12.76 | 0.41 | 0.83 | 0.53 |  | 5.35 | 1.00 | 0.18 | 0.43 |
| TMTV2.5 | 41.32 | 0.67 | 0.75 | 0.62 |  | 10.08 | 1.00 | 0.25 | 0.56 |
| TMTV30% | 14.21 | 0.89 | 0.58 | 0.66 |  | 22.83 | 0.82 | 0.43 | 0.57 |
| TMTV40% | 10.01 | 0.85 | 0.58 | 0.67 |  | 13.61 | 0.82 | 0.43 | 0.55 |
| TMTV50% | 6.36 | 0.82 | 0.58 | 0.66 |  | 8.37 | 0.82 | 0.43 | 0.53 |
| TTLG2.5 | 236.30 | 0.67 | 0.75 | 0.62 |  | 46.95 | 1.00 | 0.21 | 0.51 |
| TTLG30% | 184.45 | 0.67 | 0.75 | 0.65 |  | 89.05 | 0.82 | 0.43 | 0.52 |
| TTLG40% | 138.00 | 0.67 | 0.75 | 0.65 |  | 66.95 | 0.82 | 0.43 | 0.51 |
| TTLG50% | 48.90 | 0.74 | 0.67 | 0.63 |  | 252.05 | 0.91 | 0.29 | 0.49 |
| SLR | 0.75 | 0.59 | 0.67 | 0.51 |  | 0.85 | 0.64 | 0.82 | 0.69 |
| dNLR | 2.02 | 0.81 | 0.58 | 0.66 |  | 2.30 | 0.73 | 0.54 | 0.57 |

ROC, receiver operating characteristic; PFS; progression-free survival; OS, overall survival; Sen, sensitivity; Spe, specificity; AUC, area under the curve; SUV_max_, maximum standardized uptake value; SUV_peak_, peak standardized uptake value; TMTV, total metabolic tumor volume; TTLG, total total lesion glycolysis; SLR, spleen-to-liver ratio; dNLR, derived neutrophil-to-lymphocyte ratio

**Supplementary tables 3 (STE scanner only, n=27).**

3-1. Cut-off values of parameters by ROC curve analysis

| Variables | PFS | | | |  | OS | | | |
| --- | --- | --- | --- | --- | --- | --- | --- | --- | --- |
|  | Cut-off | Sen | Spe | AUC |  | Cut-off | Sen | Spe | AUC |
| SUV_max_ | 13.07 | 0.53 | 0.50 | 0.41 |  | 10.34 | 0.83 | 0.29 | 0.48 |
| SUV_peak_ | 9.57 | 0.53 | 0.50 | 0.51 |  | 5.47 | 1.00 | 0.24 | 0.46 |
| TMTV40% | 10.83 | 0.84 | 0.63 | 0.71 |  | 13.61 | 0.83 | 0.43 | 0.48 |
| TTLG40% | 138.00 | 0.63 | 0.88 | 0.68 |  | 66.95 | 0.83 | 0.43 | 0.44 |
| SLR | 0.75 | 0.47 | 0.75 | 0.51 |  | 0.78 | 0.67 | 0.76 | 0.69 |
| dNLR | 2.02 | 0.89 | 0.63 | 0.67 |  | 2.45 | 0.83 | 0.57 | 0.59 |

ROC, receiver operating characteristic; PFS; progression-free survival; OS, overall survival; Sen, sensitivity; Spe, specificity; AUC, area under the curve; SUV_max_, maximum standardized uptake value; SUV_peak_, peak standardized uptake value; TMTV, total metabolic tumor volume; TTLG, total total lesion glycolysis; SLR, spleen-to-liver ratio; dNLR, derived neutrophil-to-lymphocyte ratio

3-2. Univariable analysis for PFS by Cox proportional hazard model

| Variables | HR (95% CI) | *p*-value |
| --- | --- | --- |
| SUV_max_ (<13.07 vs. ≥13.07) | 0.61 (0.23 - 1.58) | 0.305 |
| SUV_peak_ (<9.57 vs. ≥9.57) | 0.61 (0.23 - 1.58) | 0.305 |
| TMTV (<10.83 vs. ≥10.83) | 3.28 (0.94 - 11.42) | 0.062 |
| TTLG (<138.00 vs. ≥138.00) | 2.80 (1.06 - 7.38) | 0.037* |
| SLR (<0.75 vs. ≥0.75) | 1.43 (0.57 - 3.62) | 0.447 |
| Age | 0.97 (0.95 - 1.00) | 0.058 |
| Sex (Female vs. Male) | 0.50 (0.18 - 1.36) | 0.174 |
| Stage (Recurrence vs. Advanced) | 0.83 (0.26 - 2.61) | 0.746 |
| Type of immunotherapy (Single vs. Combined) | 1.09 (0.41 - 2.91) | 0.869 |
| dNLR (<2.02 vs. ≥2.02) | 5.61 (1.26 - 24.98) | 0.023* |
| Group (TMTV + dNLR) |  | 0.052 |
| Group 1 vs. Group 2 | 2.92 (0.30 - 28.32) | 0.355 |
| Group 1 vs. Group 3 | 8.17 (1.06 - 63.03) | 0.044* |

PFS, progression-free survival; HR, hazard ratio; CI, confidence interval; SUV_max_, maximum standardized uptake value; SUV_peak_, peak standardized uptake value; TMTV, total metabolic tumor volume; TTLG, total total lesion glycolysis; SLR, spleen-to-liver ratio; dNLR, derived neutrophil-to-lymphocyte ratio; Group 1, low TMTV and low dNLR; Group 2, high TMTV or high dNLR; Group 3, high TMTV and high dNLR, *, statistically significant

3-3. Multivariable analysis for PFS by Cox proportional hazard model

| Variables | HR (95% CI) | *p*-value |
| --- | --- | --- |
| TMTV (<10.83 vs. ≥10.83) | 1.83 (0.48 - 6.98) | 0.379 |
| Age | 0.99 (0.96 - 1.01) | 0.321 |
| dNLR (<2.02 vs. ≥2.02) | 3.85 (1.78 - 19.02) | 0.098 |
| TTLG (<138.00 vs. ≥138.00) | 1.37 (0.47 - 4.07) | 0.566 |
| Age | 0.98 (0.96 - 1.01) | 0.269 |
| dNLR (<2.02 vs. ≥2.02) | 3.98 (0.74 - 21.37) | 0.108 |
| Group (TMTV + dNLR)* |  | 0.102 |
| Group 1 vs. Group 2 | 2.89 (0.30 - 28.00) | 0.360 |
| Group 1 vs. Group 3 | 7.07 (0.89 - 56.09) | 0.064 |
| Age | 0.99 (0.96 - 1.01) | 0.310 |

PFS, progression-free survival; HR, hazard ratio; CI, confidence interval; TMTV, total metabolic tumor volume; dNLR, derived neutrophil-to-lymphocyte ratio; TTLG, total total lesion glycolysis; *, analysis excluding TMTV, TTLG, and dNLR to avoid multicollinearity

3-4. Univariable analysis for OS by Cox proportional hazard model

| Variables | HR (95% CI) | *p*-value |
| --- | --- | --- |
| SUV_max_ (<10.34 vs. ≥10.34) | 1.70 (0.19 - 15.22) | 0.637 |
| SUV_peak_ (<5.47 vs. ≥5.47) | 30.06 (0.00 - 241039.59) | 0.458 |
| TMTV (<13.61 vs. ≥13.61) | 3.04 (0.35 - 26.55) | 0.314 |
| TTLG (<66.95 vs. ≥66.95) | 3.04 (0.35 - 26.55) | 0.314 |
| SLR (<0.78 vs. ≥0.78) | 3.76 (0.68 - 20.77) | 0.129 |
| Age | 1.00 (0.94 - 1.07) | 0.981 |
| Sex (Female vs. Male) | 0.28 (0.06 - 1.41) | 0.123 |
| Stage (Recurrence vs. Advanced) | 0.54 (0.10 - 3.06) | 0.487 |
| Type of immunotherapy (Single vs. Combined) | 0.91 (0.17 - 5.00) | 0.913 |
| dNLR (<2.45 vs. ≥2.45) | 6.33 (0.73 - 54.57) | 0.093 |
| Group (TMTV + dNLR) |  | 0.432 |
| Group 1 vs. Group 2 | 1.83 (0.00 - 380.34) | 0.975 |
| Group 1 vs. Group 3 | 4.19 (0.48 - 36.65) | 0.195 |

OS, overall survival; HR, hazard ratio; CI, confidence interval; SUV_max_, maximum standardized uptake value; SUV_peak_, peak standardized uptake value; TMTV, total metabolic tumor volume; TTLG, total total lesion glycolysis; SLR, spleen-to-liver ratio; dNLR, derived neutrophil-to-lymphocyte ratio; Group 1, low TMTV and low dNLR; Group 2, high TMTV or high dNLR; Group 3, high TMTV and high dNLR

**Supplementary tables 4 (Immunotherapy single regimen only, n=16).**

4-1. Cut-off values of parameters by ROC curve analysis

| Variables | PFS | | | |  | OS | | | |
| --- | --- | --- | --- | --- | --- | --- | --- | --- | --- |
|  | Cut-off | Sen | Spe | AUC |  | Cut-off | Sen | Spe | AUC |
| SUV_max_ | 12.15 | 0.60 | 0.50 | 0.42 |  | 15.33 | 0.40 | 0.64 | 0.38 |
| SUV_peak_ | 8.86 | 0.50 | 0.50 | 0.42 |  | 9.65 | 0.40 | 0.64 | 0.36 |
| TMTV40% | 29.87 | 0.50 | 0.67 | 0.53 |  | 15.20 | 0.80 | 0.45 | 0.56 |
| TTLG40% | 69.15 | 0.70 | 0.50 | 0.55 |  | 352.80 | 0.40 | 0.73 | 0.53 |
| SLR | 0.73 | 0.60 | 0.50 | 0.43 |  | 0.77 | 0.80 | 0.73 | 0.78 |
| dNLR | 2.30 | 0.80 | 0.67 | 0.60 |  | 3.26 | 0.60 | 0.64 | 0.58 |

ROC, receiver operating characteristic; PFS; progression-free survival; OS, overall survival; Sen, sensitivity; Spe, specificity; AUC, area under the curve; SUV_max_, maximum standardized uptake value; SUV_peak_, peak standardized uptake value; TMTV, total metabolic tumor volume; TTLG, total total lesion glycolysis; SLR, spleen-to-liver ratio; dNLR, derived neutrophil-to-lymphocyte ratio

4-2. Univariable analysis for PFS by Cox proportional hazard model

| Variables | HR (95% CI) | *p*-value |
| --- | --- | --- |
| SUV_max_ (<12.15 vs. ≥12.15) | 1.00 (0.27 - 3.74) | 0.997 |
| SUV_peak_ (<8.86 vs. ≥8.86) | 0.98 (0.26 - 3.72) | 0.972 |
| TMTV (<29.87 vs. ≥29.87) | 1.51 (0.40 - 5.64) | 0.544 |
| TTLG (<69.15 vs. ≥69.15) | 1.43 (0.36 - 5.76) | 0.615 |
| SLR (<0.73 vs. ≥0.73) | 1.51 (0.39 - 5.79) | 0.548 |
| Age | 0.94 (0.90 - 0.99) | 0.026* |
| Sex (Female vs. Male) | 0.71 (0.14 - 3.53) | 0.679 |
| Stage (Recurrence vs. Advanced) | 0.67 (0.18 - 2.49) | 0.544 |
| dNLR (<2.30 vs. ≥2.30) | 102.82 (0.30 - 35289.20) | 0.120 |
| Group (TMTV + dNLR) |  | 0.125 |
| Group 1 vs. Group 2 | 5.83 (0.64 - 52.96) | 0.117 |
| Group 1 vs. Group 3 | 10.72 (1.10 - 104.78) | 0.041* |

PFS, progression-free survival; HR, hazard ratio; CI, confidence interval; SUV_max_, maximum standardized uptake value; SUV_peak_, peak standardized uptake value; TMTV, total metabolic tumor volume; TTLG, total total lesion glycolysis; SLR, spleen-to-liver ratio; dNLR, derived neutrophil-to-lymphocyte ratio; Group 1, low TMTV and low dNLR; Group 2, high TMTV or high dNLR; Group 3, high TMTV and high dNLR, *, statistically significant

4-3. Multivariable analysis for PFS by Cox proportional hazard model

| Variables | HR (95% CI) | *p*-value |
| --- | --- | --- |
| TMTV (<29.87 vs. ≥29.87) | 0.70 (0.16 - 3.16) | 0.644 |
| Age | 1.00 (0.94 - 1.07) | 0.943 |
| dNLR (<2.30 vs. ≥2.30) | 350516.53 (0.00 - 124000000.00) | 0.937 |
| TTLG (<69.15 vs. ≥69.15) | 1.45 (0.30 - 7.03) | 0.644 |
| Age | 0.99 (0.92 - 1.07) | 0.743 |
| dNLR (<2.30 vs. ≥2.30) | 240890.14 (0.00 - 8075000000.00) | 0.939 |
| Group (TMTV + dNLR)* |  | 0.269 |
| Group 1 vs. Group 2 | 3.31 (0.30 - 36.88) | 0.331 |
| Group 1 vs. Group 3 | 6.67 (0.62 - 71.63) | 0.117 |
| Age | 0.95 (0.89 - 1.02) | 0.135 |

PFS, progression-free survival; HR, hazard ratio; CI, confidence interval; TMTV, total metabolic tumor volume; dNLR, derived neutrophil-to-lymphocyte ratio; TTLG, total total lesion glycolysis; *, analysis excluding TMTV, TTLG, and dNLR to avoid multicollinearity

4-4. Univariable analysis for OS by Cox proportional hazard model

| Variables | HR (95% CI) | *p*-value |
| --- | --- | --- |
| SUV_max_ (<15.33 vs. ≥15.33) | 1.33 (0.22 - 7.97) | 0.757 |
| SUV_peak_ (<9.65 vs. ≥9.65) | 1.33 (0.22 - 7.97) | 0.757 |
| TMTV (<15.20 vs. ≥15.20) | 3.08 (0.34 - 27.70) | 0.316 |
| TTLG (<352.80 vs. ≥352.80) | 1.73 (0.29 - 10.38) | 0.551 |
| SLR (<0.77 vs. ≥0.77) | 6.62 (0.73 - 59.60) | 0.092 |
| Age | 0.99 (0.93 - 1.05) | 0.688 |
| Sex (Female vs. Male) | 0.60 (0.07 - 5.41) | 0.651 |
| Stage (Recurrence vs. Advanced) | 2.73 (0.30 - 24.63) | 0.371 |
| dNLR (<3.26 vs. ≥3.26) | 2.51 (0.42 - 15.17) | 0.315 |
| Group (TMTV + dNLR) |  | 0.444 |
| Group 1 vs. Group 2 | 1.26 (0.08 - 20.48) | 0.871 |
| Group 1 vs. Group 3 | 3.57 (0.37 - 34.66) | 0.273 |

OS, overall survival; HR, hazard ratio; CI, confidence interval; SUV_max_, maximum standardized uptake value; SUV_peak_, peak standardized uptake value; TMTV, total metabolic tumor volume; TTLG, total total lesion glycolysis; SLR, spleen-to-liver ratio; dNLR, derived neutrophil-to-lymphocyte ratio; Group 1, low TMTV and low dNLR; Group 2, high TMTV or high dNLR; Group 3, high TMTV and high dNLR

**Supplementary tables 5 (Immunotherapy combined regimen only, n=23).**

5-1. Cut-off values of parameters by ROC curve analysis

| Variables | PFS | | | |  | OS | | | |
| --- | --- | --- | --- | --- | --- | --- | --- | --- | --- |
|  | Cut-off | Sen | Spe | AUC |  | Cut-off | Sen | Spe | AUC |
| SUV_max_ | 13.07 | 0.65 | 0.50 | 0.47 |  | 13.07 | 0.67 | 0.41 | 0.50 |
| SUV_peak_ | 12.76 | 0.47 | 0.83 | 0.57 |  | 9.57 | 0.67 | 0.47 | 0.48 |
| TMTV40% | 9.08 | 0.82 | 0.83 | 0.76 |  | 12.67 | 0.83 | 0.47 | 0.53 |
| TTLG40% | 102.95 | 0.77 | 0.83 | 0.73 |  | 102.95 | 0.83 | 0.47 | 0.50 |
| SLR | 0.75 | 0.65 | 0.83 | 0.58 |  | 0.85 | 0.67 | 0.88 | 0.64 |
| dNLR | 1.74 | 0.94 | 0.67 | 0.76 |  | 2.45 | 0.67 | 0.65 | 0.56 |

ROC, receiver operating characteristic; PFS; progression-free survival; OS, overall survival; Sen, sensitivity; Spe, specificity; AUC, area under the curve; SUV_max_, maximum standardized uptake value; SUV_peak_, peak standardized uptake value; TMTV, total metabolic tumor volume; TTLG, total total lesion glycolysis; SLR, spleen-to-liver ratio; dNLR, derived neutrophil-to-lymphocyte ratio

5-2. Univariable analysis for PFS by Cox proportional hazard model

| Variables | HR (95% CI) | *p*-value |
| --- | --- | --- |
| SUV_max_ (<13.07 vs. ≥13.07) | 0.91 (0.33 - 2.51) | 0.855 |
| SUV_peak_ (<12.76 vs. ≥12.76) | 1.04 (0.38 - 2.81) | 0.942 |
| TMTV (<9.08 vs. ≥9.08) | 4.16 (1.17 - 14.85) | 0.028* |
| TTLG (<102.95 vs. ≥102.95) | 3.24 (1.03 - 10.16) | 0.044* |
| SLR (<0.75 vs. ≥0.75) | 1.69 (0.54 - 5.24) | 0.364 |
| Age | 0.98 (0.95 - 1.00) | 0.088 |
| Sex (Female vs. Male) | 0.44 (0.14 - 1.40) | 0.165 |
| Stage (Recurrence vs. Advanced) | 0.99 (0.31 - 3.16) | 0.981 |
| dNLR (<1.74 vs. ≥1.74) | 6.81 (0.89 - 52.10) | 0.065 |
| Group (TMTV + dNLR) |  | 0.920 |
| Group 1 vs. Group 2 | 97340.90 (0.00 - 246500000.00) | 0.929 |
| Group 1 vs. Group 3 | 122835.248 (0.00 - 31060000000.00) | 0.928 |

PFS, progression-free survival; HR, hazard ratio; CI, confidence interval; SUV_max_, maximum standardized uptake value; SUV_peak_, peak standardized uptake value; TMTV, total metabolic tumor volume; TTLG, total total lesion glycolysis; SLR, spleen-to-liver ratio; dNLR, derived neutrophil-to-lymphocyte ratio; Group 1, low TMTV and low dNLR; Group 2, high TMTV or high dNLR; Group 3, high TMTV and high dNLR, *, statistically significant

5-3. Multivariable analysis for PFS by Cox proportional hazard model

| Variables | HR (95% CI) | *p*-value |
| --- | --- | --- |
| TMTV (<9.08 vs. ≥9.08) | 2.17 (0.51 - 9.32) | 0.298 |
| Age | 0.99 (0.96 - 1.01) | 0.300 |
| dNLR (<1.74 vs. ≥1.74) | 3.57 (0.36 - 35.80) | 0.279 |
| TTLG (<102.95 vs. ≥102.95) | 1.72 (0.49 - 6.07) | 0.402 |
| Age | 0.99 ( 0.96 - 1.01) | 0.274 |
| dNLR (<1.74 vs. ≥1.74) | 4.30 (0.47 - 39.61) | 0.198 |
| Group (TMTV + dNLR)* |  | 0.982 |
| Group 1 vs. Group 2 | 103072.14 (0.00 - 154200000.00) | 0.931 |
| Group 1 vs. Group 3 | 114078.06 (0.00 - 17050000000.00) | 0.930 |
| Age | 0.98 ( 0.96 - 1.01) | 0.250 |

PFS, progression-free survival; HR, hazard ratio; CI, confidence interval; TMTV, total metabolic tumor volume; dNLR, derived neutrophil-to-lymphocyte ratio; TTLG, total total lesion glycolysis; *, analysis excluding TMTV, TTLG, and dNLR to avoid multicollinearity

5-4. Univariable analysis for OS by Cox proportional hazard model

| Variables | HR (95% CI) | *p*-value |
| --- | --- | --- |
| SUV_max_ (<13.07 vs. ≥13.07) | 0.95 (0.17 - 5.25) | 0.956 |
| SUV_peak_ (<9.57 vs. ≥9.57) | 0.99 (0.18 - 5.45) | 0.986 |
| TMTV (<12.67 vs. ≥12.67) | 2.79 (0.32 - 24.17) | 0.351 |
| TTLG (<102.95 vs. ≥102.95) | 2.79 (0.32 - 24.17) | 0.351 |
| SLR (<0.85 vs. ≥0.85) | 5.63 (1.00 - 31.84) | 0.051 |
| Age | 0.96 (0.90 - 1.03) | 0.253 |
| Sex (Female vs. Male) | 0.32 (0.05 - 1.93) | 0.215 |
| Stage (Recurrence vs. Advanced) | 0.26 (0.03 - 1.97) | 0.192 |
| dNLR (<2.45 vs. ≥2.45) | 3.48 (0.63 - 19.34) | 0.154 |
| Group (TMTV + dNLR) |  | 0.361 |
| Group 1 vs. Group 2 | 1.29 (0.08 - 21.00) | 0.859 |
| Group 1 vs. Group 3 | 3.91 (0.43 - 35.48) | 0.225 |

OS, overall survival; HR, hazard ratio; CI, confidence interval; SUV_max_, maximum standardized uptake value; SUV_peak_, peak standardized uptake value; TMTV, total metabolic tumor volume; TTLG, total total lesion glycolysis; SLR, spleen-to-liver ratio; dNLR, derived neutrophil-to-lymphocyte ratio; Group 1, low TMTV and low dNLR; Group 2, high TMTV or high dNLR; Group 3, high TMTV and high dNLR
